# Science in action for safer food: World Food Safety Day 2025

**DOI:** 10.5365/wpsar.2025.16.2.1294

**Published:** 2025-06-07

**Authors:** Jessica Kayamori Lopes, Syed Moazzem Hossain, Simone Moraes Raszl

**Affiliations:** a World Health Organization Regional Office for the Western Pacific, Manila, Philippines.; b World Health Organization, Geneva, Switzerland.

The global public health community observes the annual World Food Safety Day on 7 June under the auspices of the Food and Agriculture Organization (FAO) and the World Health Organization (WHO). The 2025 theme, “Food Safety: Science in Action,” emphasizes the role of science in identifying food safety hazards, preventing foodborne illness and guiding decision-making from farm to fork.

Since its launch in 2019, World Food Safety Day has become a vital global platform for raising awareness and mobilizing action on food safety. Its objective is to engage governments, the private sector, academia and civil society in promoting food safety education, policy dialogue and community involvement. These efforts seek to prevent, detect and manage foodborne risks, contributing to food security, public health, economic growth, agricultural development, market access, tourism and sustainable development. World Food Safety Day has strengthened cross-sector collaboration, encouraged the enhancement of national food safety systems and supported the adoption of Codex Alimentarius international food standards through science-based approaches.

This year, FAO and WHO have released a World Food Safety Day 2025 campaign launch video, communications tools, campaign materials and more to help countries and stakeholders organize awareness activities such as educational campaigns, exhibitions, quizzes and webinars. (*1*) These events aim to engage the public, promote cross-sector collaboration and emphasize that safe food is a shared responsibility.

Food safety is crucial for public health, food security and economic development and is a shared responsibility across the supply chain – from producers to consumers. Science-based practices and established standards are essential to prevent food contamination and ensure food safety. Scientific evidence identifies hazards, informs risk management, and guides policy-makers, businesses and the public. It also supports evidence-based policy decisions, promotes good hygiene practices in food operations, and encourages safer consumer behaviours. (*2*) For example, scientific risk assessments of microbial contamination in fresh produce have led to safety standards, improved market hygiene and better public handling practices in the Western Pacific Region. Without science, food safety maintenance in global supply chains would be impossible.

According to WHO, 125 million people fall ill and over 50 000 die annually in the Region from unsafe food. Children aged < 5 years are particularly affected, accounting for 30% of foodborne illness cases and 7000 deaths each year. (*3*) These statistics underscore the need for stronger food safety systems, especially in low- and middle-income countries with inadequate regulation, poor infrastructure, limited surveillance and low consumer awareness. (*4*)

In the Region, the coexistence of diverse food production systems, reliance on food imports and traditional food markets together with rapid urbanization necessitates the integration of scientific advances into food safety policies and practices. Governments are encouraged to invest in laboratory capacity, food safety education, early warning systems and regional information exchange. The private sector, academia and civil society also play vital roles in advancing and applying scientific knowledge. (*5*)

The Regional Framework for Action on Food Safety in the Western Pacific, (*5*) endorsed by the WHO Regional Committee in 2017, recognizes the evolving landscape of food safety and redefines strategies for strengthening national food safety systems. It emphasizes building public trust and confidence, while promoting strategic actions that foster leadership, partnerships, capacity and resource mobilization, all of which are key enablers for advancing food safety. Aimed at national food safety authorities, the Framework offers guidance on strategic planning and a science-based, step-by-step approach to manage food safety risks effectively and respond to incidents and emergencies.

Ultimately, food safety is a shared commitment to health, equity and sustainability. On World Food Safety Day 2025, WHO reaffirms the importance of science and evidence-based data for prevention, protection and progress. By translating scientific knowledge into action, WHO can strengthen food safety systems, reduce the incidence of foodborne illness and contribute to a healthier future for all.
